# Cholesterol and host cell surface proteins contribute to cell-cell fusion induced by the *Burkholderia* type VI secretion system 5

**DOI:** 10.1371/journal.pone.0185715

**Published:** 2017-10-03

**Authors:** Liam Whiteley, Maria Haug, Kristina Klein, Matthias Willmann, Erwin Bohn, Salvatore Chiantia, Sandra Schwarz

**Affiliations:** 1 Interfaculty Institute of Microbiology and Infection Medicine, University of Tuebingen, Tuebingen, Germany; 2 Department of Biochemistry, Potsdam University, Potsdam, Germany; Centre National de la Recherche Scientifique, Aix-Marseille Université, FRANCE

## Abstract

Following escape into the cytoplasm of host cells, *Burkholderia pseudomallei* and the related species *Burkholderia thailandensis* employ the type VI secretion system 5 (T6SS-5) to induce plasma membrane fusion with an adjacent host cell. This process leads to the formation of multinucleated giant cells and facilitates bacterial access to an uninfected host cell in a direct manner. Despite its importance in virulence, the mechanism of the T6SS-5 and the role of host cell factors in cell-cell fusion remain elusive. To date, the T6SS-5 is the only system of bacterial origin known to induce host-cell fusion. To gain insight into the nature of T6SS-5-stimulated membrane fusion, we investigated the contribution of cholesterol and proteins exposed on the host cell surface, which were shown to be critically involved in virus-mediated giant cell formation. In particular, we analyzed the effect of host cell surface protein and cholesterol depletion on the formation of multinucleated giant cells induced by *B*. *thailandensis*. Acute protease treatment of RAW264.7 macrophages during infection with *B*. *thailandensis* followed by agarose overlay assays revealed a strong reduction in the number of cell-cell fusions compared with EDTA treated cells. Similarly, proteolytic treatment of specifically infected donor cells or uninfected recipient cells significantly decreased multinucleated giant cell formation. Furthermore, modulating host cell cholesterol content by acute cholesterol depletion from cellular membranes by methyl- β-cyclodextrin treatment or exogenous addition of cholesterol impaired the ability of *B*. *thailandensis* to induce cell-cell fusions. The requirement of physiological cholesterol levels suggests that the membrane organization or mechanical properties of the lipid bilayer influence the fusion process. Altogether, our data suggest that membrane fusion induced by *B*. *pseudomallei* and *B*. *thailandensis* involves a complex interplay between the T6SS-5 and the host cell.

## Introduction

*Burkholderia pseudomallei* causes the potentially fatal disease melioidosis in humans and animals [1]. Central to the pathogenesis of *B*. *pseudomallei* is its ability to adopt a facultative intracellular life style involving lysis of the vacuolar membrane and escape into the cytoplasm of the host cell [2, 3]. After gaining access to the cytosol, the bacteria impose a cytopathic effect upon host cells by fusing the infected cell with one or more neighboring cells, leading to the formation of multinucleated giant cells (MNGCs) [4]. While the ability to induce cell-cell fusions is unique for bacteria it is a common trait among enveloped viruses such as human immunodeficiency, measles and herpes virus [5–7]. The induction of host cell fusions is proposed to facilitate dissemination of the virus and escape from immune defenses, such as neutralizing antibodies [8, 9]. The same might apply for *B*. *pseudomallei-*stimulated MNGC formation, for which the biological function has yet to be determined. The type VI secretion system 5 (T6SS-5; also named T6SS1) present in *B*. *pseudomallei* and the closely related species *Burkholderia thailandensis* is specifically expressed inside host cells and is essential for the induction of MNGCs [10–14]. Importantly, the T6SS-5 is a major virulence factor in animal models of infection [10, 15, 16]. This finding suggests a central role of host membrane fusion during *B*. *pseudomallei* and *B*. *thailandensis* infection although the T6SS-5 still awaits full functional characterization.

A recent global secretome analysis of *B*. *thailandensis* wild type and a mutant producing a disrupted T6SS-5 yielded one candidate protein secreted by the T6SS-5 with potential effector activity [12]. The protein, VgrG-5, belongs to the group of specialized VgrG proteins that contain a variable C-terminal extension in addition to the ubiquitous domains that form the needle of the T6S apparatus. The C-terminal domain of VgrG-5 is required for MNGC formation and initiation of a lethal infection in mice but not for secretory activity of the T6SS-5 [12, 13]. At present, work on the VgrG-5 protein did not result in the identification of the host cell target of the T6SS-5.

To date, proteins of bacterial origin that mediate host cell fusions have not been described. Likewise, an injection device such as the T6SS-5 is a novel player in the field of cell-cell fusion. The plasma membrane rearrangements induced by the T6SS-5 are unknown. In addition, the role of proteins located on the surface of the host cell in MNGC formation has not been investigated. Surface-exposed components of the host cell such as membrane proteins are critically involved in membrane fusions induced by several viruses by specifically activating viral fusion proteins and determining viral host cell tropism [17–19]. Furthermore, the specific composition of the lipid membranes influences the fusion process. In particular, cholesterol, an abundant component of plasma membranes, can significantly affect membrane fusion by i) altering the mechanical properties of the lipid bilayer, ii) direct interactions with fusion proteins or iii) formation of cholesterol rich membrane rafts clustering fusion proteins and host receptors [20–22]. In this study, we sought to gain insight into the mechanism of T6SS-5-mediated membrane fusion by investigating the role of host cell factors. We found that host cell surface proteins and cholesterol contribute to MNGC formation induced by *B*. *thailandensis*.

## Results

### Acute depletion of surface proteins from host cells during infection with *B*. *thailandensis* decreases MNGC formation

**Fig 1:**
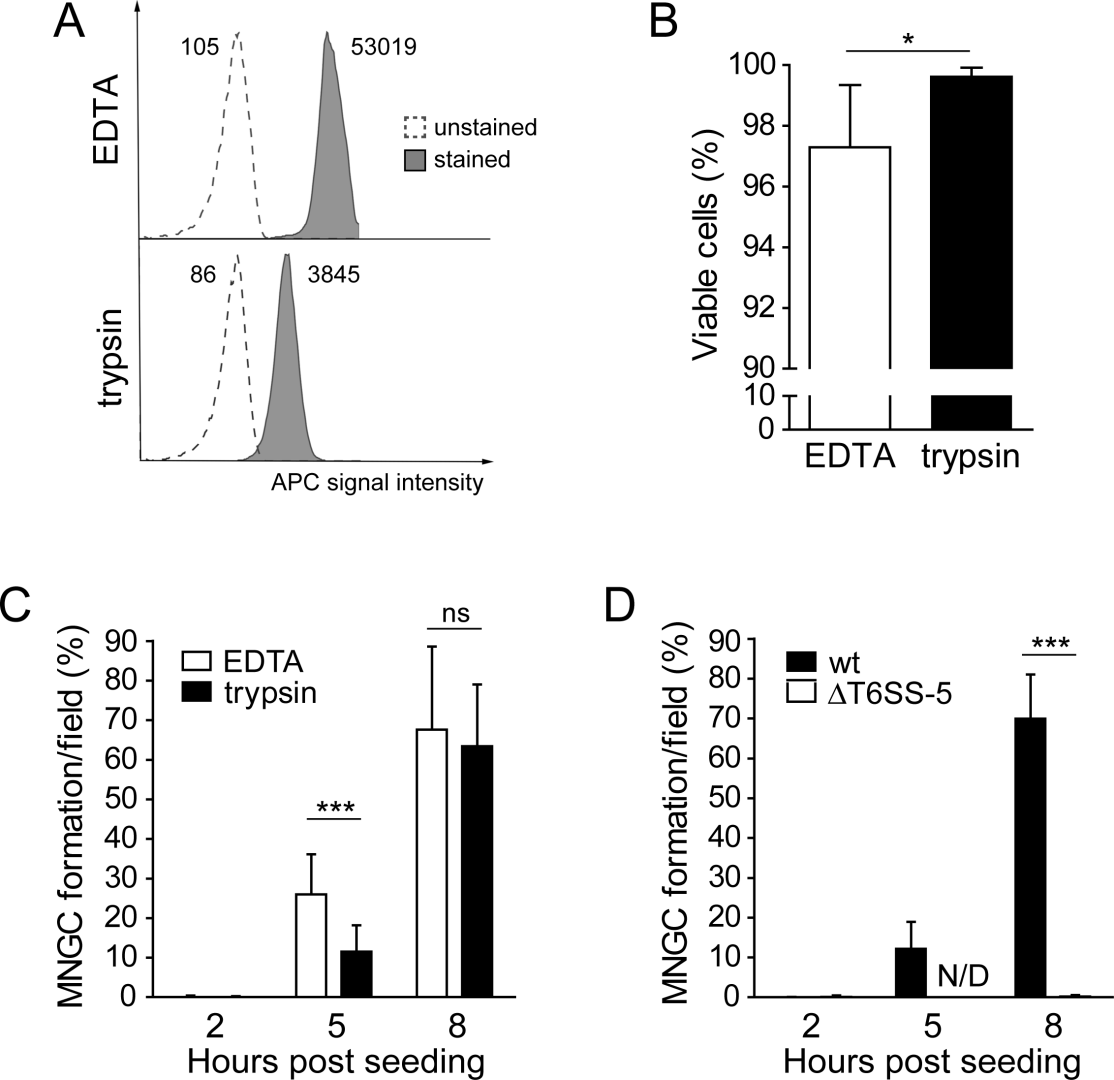
Acute depletion of host cell surface proteins from RAW264.7 macrophages during infection reduces T6SS-5 stimulated cell-cell fusions. A) FACS histograms of RAW264.7 macrophages treated with 0.05% trypsin or 0.05% EDTA for 30 min at 37°C and immediately stained with α integrin β-2 (CD18) antibody conjugated to APC. Numbers in the panel indicate the mean fluorescence intensity (log scale). Representative results from one of two independent experiments are shown. B) Viability of macrophages after EDTA or trypsin treatment for 30 min at 37°C. Viability of detached cells was measured immediately after the treatment. Data are shown as mean + SD of three independent experiments performed in duplicate. *, *P* = 0.0458 (Welch’s t-test)/effect size: 0.346 (Glass’s Δ). C) MNGC formation of macrophages infected with *B*. *thailandensis* at an MOI of 50 for 3 h, at which point 0.05% trypsin or 0.05% EDTA was added for 30 min. Detached cells were collected and quantified and MNGC formation was determined at the indicated time points post seeding using an agarose overlay assay. Data represent the mean + SD of two independent experiments performed in duplicate. ***, *P* < 0.0001 (t-test)/effect size: 1.609 (Hedges’ *g*); ns, not significant, *P* value = 0.468 (Mann Whitney test)/effect size: 0.229 (Mann Whitney *r*). D) Infection of macrophages with *B*. *thailandensis* wild type (wt) and ΔT6SS-5 mutant at MOI 50 for 3 h and subsequent treatment with trypsin for 30 min. MNGC formation was determined of detached cells at the indicated time points using agarose overlay assays. Data are shown as mean + SD of two independent experiment performed in duplicate. ***, *P* < 0.0001 (Mann Whitney test)/effect size: 0.870 (Mann Whitney *r*); N/D, not detected.

We used the adherent murine macrophage cell line RAW264.7, which displays pronounced MNGC formation after a few hours post infection with *B*. *thailandensis* wild type. As a control, we infected macrophages with a mutant lacking an essential component of the T6SS-5, TssK-5, which we will refer to as ΔT6SS-5 [16]. This mutant is unable to induce MNGC formation in untreated RAW264.7 macrophages and other cells [23] (S1 Fig). To determine the role of host cell surface proteins in T6SS-5 mediated cell-cell fusions, macrophages infected with *B*. *thailandensis* wild type were incubated with 0.05% trypsin for 30 min at 37°C approximately 3 h before the onset of MNGC formation. Treatment of the macrophages with trypsin leads to detachment and requires agarose overlay assays to bring them back into contact with the plate surface and other macrophages for monitoring MNGC formation. To relate the effect of proteolytic treatment on host cell fusion to an appropriate control (*i*.*e*. to cells that underwent the same experimental procedure), we incubated infected macrophages with 0.05% EDTA, a treatment that also resulted in cellular detachment. Successful cleavage of surface proteins was confirmed by staining the cells with an APC-conjugated antibody against integrin β-2, an abundant surface exposed protein and subsequent analysis by flow cytometry. Trypsin treatment resulted in an approximately 14-fold reduction of mean fluorescence intensity of APC compared with EDTA treated cells indicating efficient break down of surface proteins (Fig 1A). Trypan blue staining showed that 99% of trypsin treated macrophages were viable (Fig 1B).

To analyze the effect of the depletion of proteins located on the surface of host cells on cell-cell fusion, macrophages were infected with *B*. *thailandensis* for 3 h, washed and split into two groups, one treated with trypsin and the other one with EDTA. The cells were collected, counted and seeded using agarose overlay to bring the macrophages into contact. Virtually no host cell fusions of EDTA and trypsin treated macrophages were observed 2 h after seeding (Fig 1C and S2 Fig). At 5 h post seeding, MNGC formation of cells incubated with EDTA and trypsin was detectable, which differed significantly in the extent between the two treatments. We found that *B*. *thailandensis* stimulated 26% MNGC formation in EDTA treated cells whereas proteolytic treatment reduced the number of cell-cell fusions to 12% (Fig 1C and S2 Fig). These results indicate that protein(s) located on the host cell surface contribute to the fusion process induced by *B*. *thailandensis*. MNGC formation of EDTA and trypsin treated cells was similar after an 8 h recovery from the respective treatment. This finding confirms that the observed difference in the number of cell-cell fusions at 5 h post seeding was not due to reduced viability of the macrophages treated with trypsin. Consistent with previous studies demonstrating a requirement of the T6SS-5 for MNGC formation of untreated cells, we did not observe cell-cell fusions in trypsin treated macrophages infected with *B*. *thailandensis* ΔT6SS-5 at 5 and 8 h post seeding (Fig 1D and S2 Fig).

### Contribution of surface proteins located on infected donor and uninfected recipient host cells to T6SS-5 mediated cell-cell fusions

**Fig 2:**
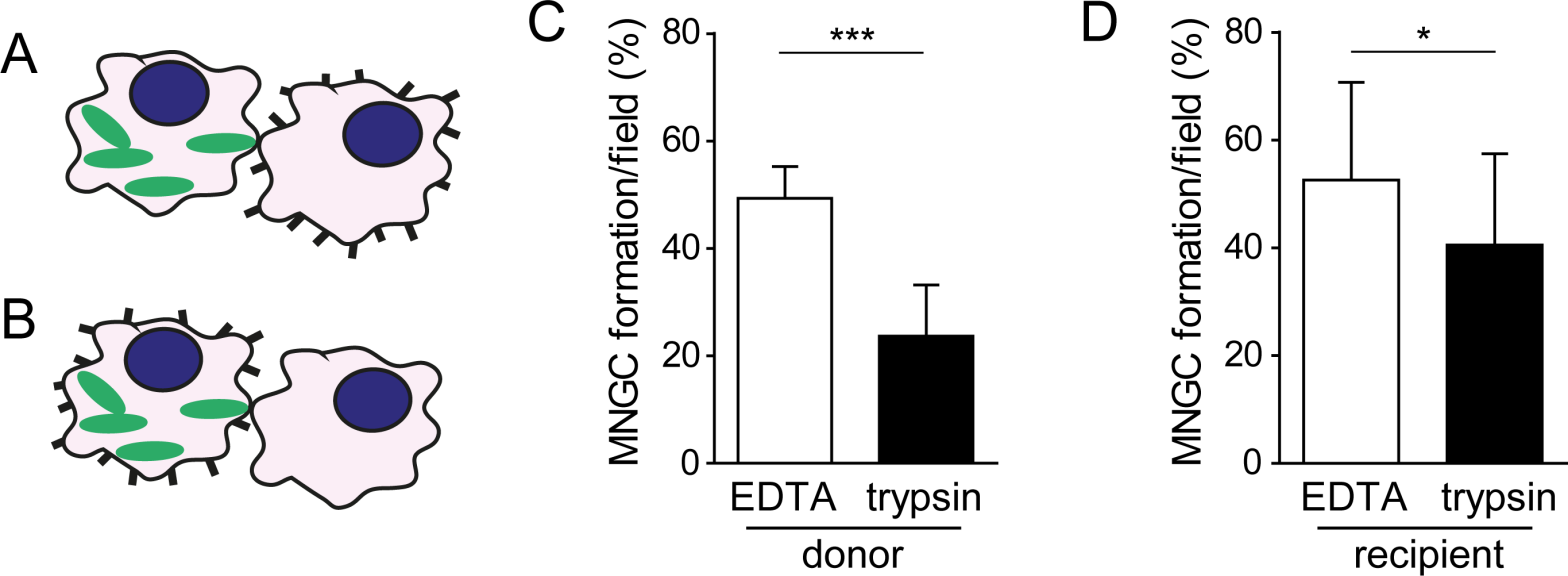
Proteins located specifically on the surface of both donor (infected) and recipient (neighboring uninfected) RAW264.7 macrophages contribute to MNGC formation induced by *B*. *thailandensis*. A) and B) Schematic depicting acute protease treatment of donor cells and recipient cells, respectively. Green shapes indicate bacteria and black bars represent surface proteins. C) Macrophages infected with *B*. *thailandensis* at MOI 33 for approximately 6 h were treated with trypsin or EDTA for 30 min. Detached cells were mixed at equal ratios with uninfected and untreated macrophages and seeded at 6×10^5^ cells per well in a 24 well plate using agarose overlay assay. After incubating the cells for 3–4 h MNGC formation was quantified. Data represent the mean + SD of two independent experiments performed in duplicate. ***, *P* < 0.0001 (t-test)/effect size: 3.226 (Hedges’ *g*). D) Macrophages were infected with *B*. *thailandensis* at MOI 33 for approximately 6 h and mixed with trypsin or EDTA treated uninfected cells at equal ratios. The cells were seeded in a 24 well plate at 6×10^5^ cells per well and brought into contact through agarose overlay. Cell-cell fusions were calculated at 3–4 h post seeding. Data represent the mean + SD of three independent experiments performed in duplicate. *, *P* = 0.0463 (t-test)/effect size: 0.688 (Hedges’ *g*).

Having found that protease treatment of all host cells impairs T6SS-5 mediated membrane fusion we next determined whether host proteins located specifically on the infected (donor) cell or the uninfected neighboring (recipient) cell contribute to MNGC formation (Fig 2A and 2B, respectively). The involvement of surface proteins of donor cells was analyzed by infecting macrophages with *B*. *thailandensis* at MOI 33 for 6 h and subsequently treating them with trypsin or EDTA. These donor cells were mixed at equal ratios with uninfected and untreated cells and seeded using agarose overlay assays. A 49% and 24% MNGC formation efficiency was observed for EDTA and trypsin treated donor cells, respectively, when mixed with untreated recipient cells for approximately 3–4 h (Fig 2C and S3 Fig). To investigate the contribution of proteins located on the surface of recipient cells to T6SS-5 mediated host cell fusions, uninfected macrophages were incubated with trypsin or EDTA and mixed at equal ratios with cells infected with *B*. *thailandensis* at MOI 33 for 6 h but otherwise untreated. Proteolytic treatment of recipient cells with trypsin resulted in significantly reduced MNGC formation (40%) as compared with EDTA treatment (53%), although the decrease was less pronounced than that observed upon protein depletion of donor cells (Fig 2D and S3 Fig). These results suggest that trypsin sensitive surface protein(s) on both the infected and the neighboring uninfected cell might contribute to T6SS-5-stimulated cell-cell fusion.

### Acute cholesterol depletion of infected host cells impairs MNGC formation

**Fig 3:**
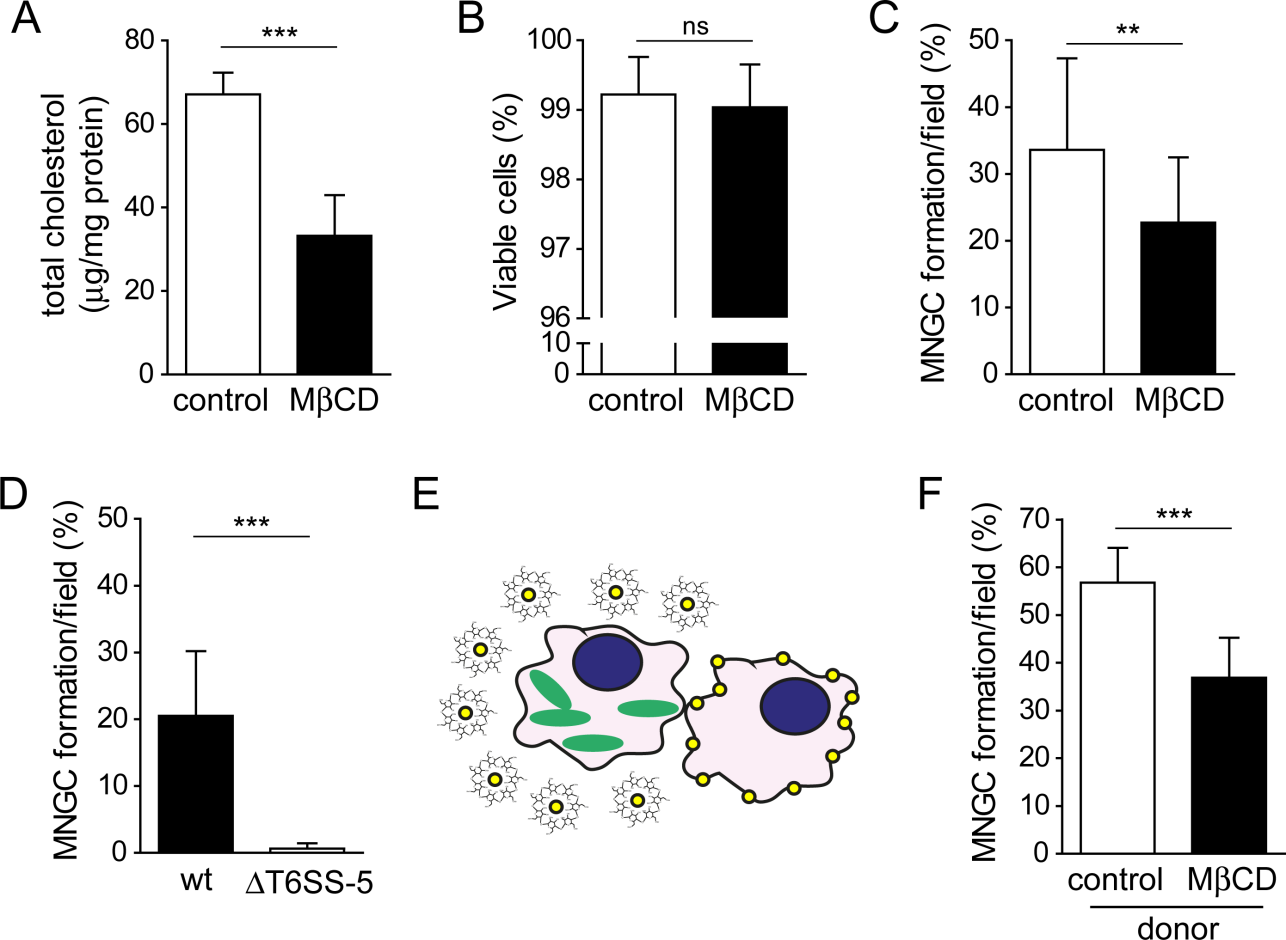
Acute treatment of RAW264.7 macrophages with the cholesterol extracting agent MβCD during infection decreases *B*. *thailandensis* mediated host cell fusions. A) Measurement of total cholesterol content of untreated (control) macrophages and cells treated with 10 mM MβCD for 1 h (MβCD) normalized to total protein content. Data are shown as mean + SD of two independent experiments performed in duplicate. ***, *P* < 0.0001 (t-test)/effect size: 4.327 (Hedges’ *g*). B) Viability of macrophages immediately after treatment with 10 mM MβCD for 1 h compared to untreated cells. Data represent mean + SD of two independent experiments performed in duplicate. ns, not significant *P* = 0.0438 (t-test)/effect size: 0.323 (Hedges’ *g*). C) MNGC formation of macrophages infected with *B*. *thailandensis* at an MOI of 17 and treated with 10 mM MβCD for 1 h shortly before the onset of MNGC formation. Shown are mean values + SD of four independent experiments performed in duplicate. **, *P* = 0.0027 (t-test)/effect size: 0.916 (Hedges’ *g*). D) MNGC formation induced by *B*. *thailandensis* wild type (wt) and ΔT6SS-5 mutant of macrophages infected and treated as described in C. Data shown are mean values + SD of two independent experiments performed in duplicate. ***, *P* < 0.0001 (Welch’s t-test)/effect size: 1.454 (Glass’s Δ). E) Schematic illustrating MβCD-based cholesterol extraction from infected donor cells. Green shape represents bacteria, yellow circles indicate cholesterol and the thin black lines denote ring-shaped heptamers formed by MβCD. F) MNGC formation of macrophages infected with *B*. *thailandensis* at MOI 33 for approximately 6 h and followed by MβCD (10 mM) treatment for 1 h. To these cells untreated and uninfected macrophages were added at equal ratios and MNGC formation was determined at 2–3 h post seeding. Data represent mean + SD of two independent experiments performed in duplicate. ***, *P* < 0.0001 (t-test)/effect size: 2.542 (Hedges’ *g*).

Methyl-β-cyclodextrin (MβCD) is a ring-shaped oligosaccharide with a hydrophobic cavity of low ability to cross membranes. MβCD has been widely used to manipulate membrane cholesterol levels to study the role of cholesterol in various membrane fusion processes and in general does not cause cellular detachment [24–26]. We incubated macrophages with 10 mM MβCD for 1 h at 37°C to sequester cholesterol from cellular membranes. Compared with untreated cells (control) this treatment resulted in a 51% decrease in total cholesterol content indicating efficient cholesterol extraction (Fig 3A). Importantly, the viability of macrophages incubated with 10 mM MβCD for 1 h was similar to that of untreated cells (Fig 3B).

Macrophages were infected with *B*. *thailandensis* at MOI 17 for approximately 8 h, which results in MNGC formation when the cells were left untreated. Acute cholesterol depletion of infected macrophages before development of MNGCs significantly reduced the number of cell-cell fusions relative to infected but untreated cells (23% and 34%, respectively) (Fig 3C and S4 Fig). This finding suggests that physiological membrane cholesterol levels are required for efficient MNGC formation. The T6SS-5 mutant is defective in induction of MNGC formation of MβCD treated cells (Fig 3D and S4 Fig). To assess if the depletion of cholesterol specifically of the infected donor cell influences MNGC formation, macrophages were infected with *B*. *thailandensis* at MOI 33 for approximately 6 h and treated with 10 mM MβCD for 1 h shortly before the onset of MNGC formation or left untreated (Fig 3E). Recipient macrophages (untreated and uninfected) were then added at equal ratios. The extraction of cholesterol from infected donor cells significantly decreased cell-cell fusions (37%) in comparison with untreated donor cells (57%) suggesting that cholesterol levels of the plasma membrane influence MNGC formation (Fig 3F and S4 Fig).

### Exogenous addition of cholesterol reduces T6SS-5-stimulated cell-cell fusions

**Fig 4:**
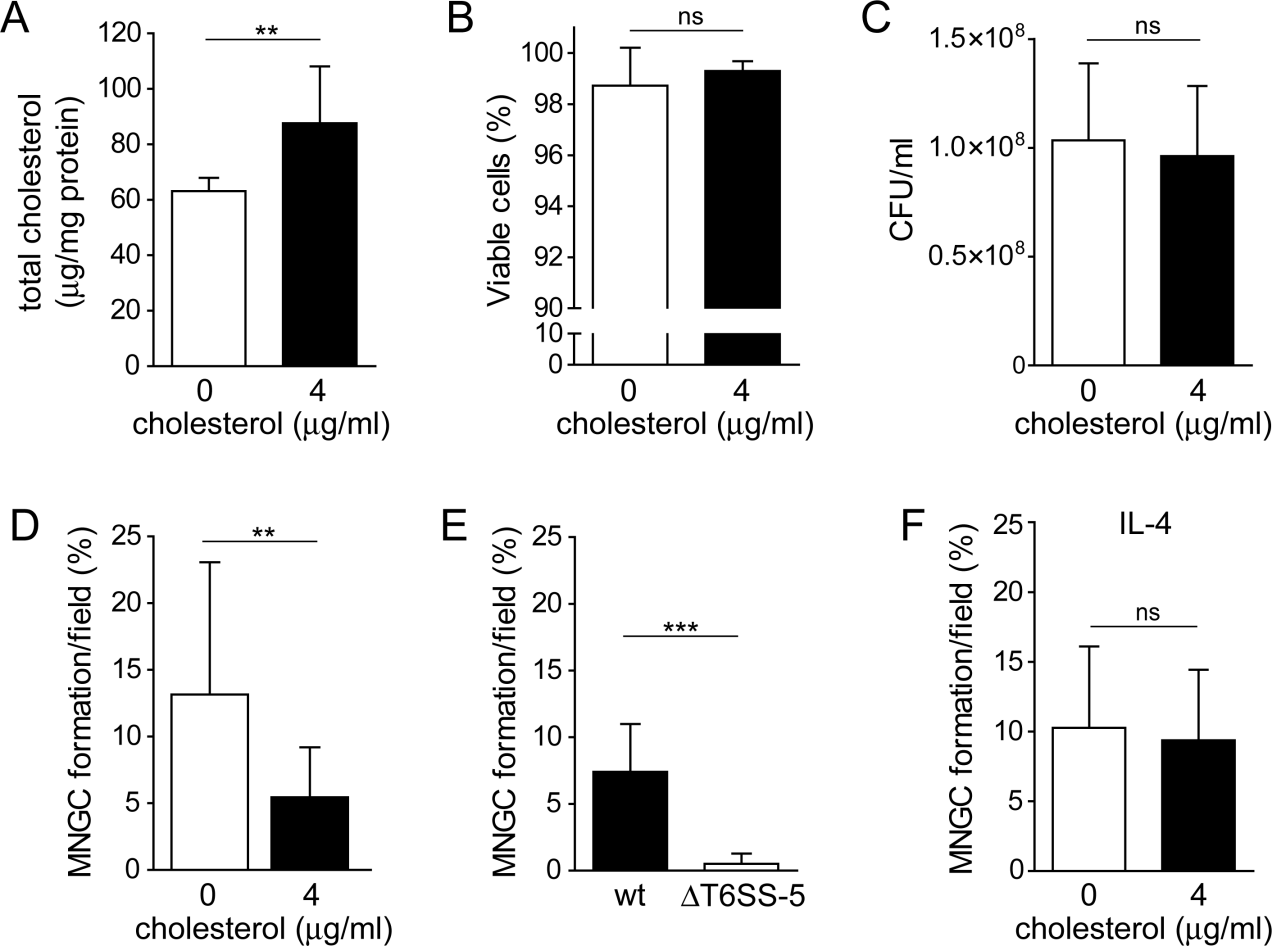
The exogenous addition of cholesterol to RAW264.7 macrophages at the time of infection with *B*. *thailandensis* reduces MNGC formation. A) Measurement of total cholesterol content of untreated (control) macrophages and cells treated with 4 μg/ml cholesterol for 10 h normalized to total cellular protein content. Shown are mean values + SD of two independent experiments performed in duplicate. **, *P* = 0.0087 (Welch’s t-test)/effect size: 5.100 (Glass’s Δ). B) Viability of macrophages after incubation with 4 μg/ml cholesterol for 10 h. Data represent mean + SD of two independent experiments performed in duplicate. ns, not significant; *P* value = 0.223 (Welch’s t-test)/effect size: 0.383 (Glass’s Δ). C) Number of *B*. *thailandensis* located inside macrophages infected at MOI 17 and simultaneously treated with cholesterol or left untreated at 10 h post-infection. Data are shown as mean values + SD of two independent experiments performed in duplicate. ns, not significant, *P* value = 0.67 (Welch’s t-test)/effect size: 2.429 (Glass’s Δ). D) MNGC formation of macrophages infected with *B*. *thailandensis* at an MOI of 17 and treated with 4 μg/ml cholesterol at the time of infection followed by a 10 h incubation period. Shown are mean values + SD of two independent experiments performed in duplicate. **, *P* = 0.0016 (Mann-Whitney test)/effect size: 0.478 (Mann-Whitney *r*). E) MNGC formation of macrophages infected with *B*. *thailandensis* wild type (wt) and ΔT6SS-5 mutant at MOI 17 and treated with cholesterol as described in D. Data represent mean values + SD of two independent experiments performed in duplicate. ***, *P* < 0.0001 (Mann-Whitney test)/effect size: 0.859 (Mann-Whitney *r*). F) MNGC formation of PMA differentiated THP-1 cells after treatment with 50 ng/ml IL-4 or 50 ng/ml IL-4 and 4 μg/ml cholesterol for three days. Data are shown as mean values + SD of two independent experiments performed in triplicate. ns, not significant, *P* value = 0.529 (t test)/effect size: 0.163 (Hedge’s *g*).

To corroborate our finding that the manipulation of cholesterol levels impairs host cell fusions induced by the T6SS-5, we next altered the composition of cellular membranes by the addition of cholesterol. For this, 4 μg/ml cholesterol was added to the cell culture medium for 10 h, which resulted in significantly increased levels of cellular cholesterol (Fig 4A). Viability assays of macrophages following the same treatment showed no cytotoxic effect (Fig 4B). The influence of additional cholesterol on MNGC formation was investigated by adding cholesterol to macrophages at the same time when infection with *B*. *thailandensis* was initiated followed by a 10 h incubation period. Thus, to control for confounding effects of cholesterol on bacteria we determined the number of intracellular bacteria after treatment of macrophages with 4 μg/ml cholesterol for 10 h. The number of colony forming units was similar between treated and untreated host cells indicating that phagocytosis and intracellular survival of *B*. *thailandensis* was not affected by the addition of cholesterol (Fig 4C). Macrophages infected with *B*. *thailandensis* in absence of exogenously added cholesterol showed 13% MNGC formation (Fig 4D). Supplementing the medium with 4 μg/ml cholesterol significantly reduced the number of cell-cell fusions as compared with untreated cells (Fig 4D and S4 Fig). Disruption of the T6SS-5 resulted in a MNGC formation defect of *B*. *thailandensis* in macrophages treated with cholesterol as described above (Fig 4E and S4 Fig).

MNGC formation of macrophages can be stimulated by certain cytokines such as IL-4 [27, 28]. We made use of this fact to test if the exogenous addition of cholesterol would affect IL-4 induced cell-cell fusions of monocytic THP-1 cells differentiated by PMA. Incubation of the cells with 50 ng/ml IL-4 alone or 50 ng/ml IL-4 and 4 μg/ml cholesterol did not result in significantly different MNGC formation (Fig 4E and S5 Fig). This observation suggests that the effect of cholesterol is specific for MNGC formation stimulated by the T6SS-5. Together, the results might indicate that a surplus of cholesterol impairs MNGC formation and that optimal levels of cholesterol are required for *B*. *thailandensis* stimulated cell-cell fusion.

## Discussion

Although the T6SS-5 is a major virulence factor in *B*. *pseudomallei* and has been known for several years its mechanism still remains enigmatic [10, 15, 29]. Progress in elucidating the molecular basis of T6SS-5 induced cell-cell fusions is hampered by the circumstance that the activity of the potential effector protein VgrG-5 is unknown and the possibility that other as yet unidentified effector proteins participate in MNGC formation. Furthermore, the T6SS-5 is the first injection device and the first system of bacterial origin described to be involved in mediating membrane fusions. Thus, information on related systems to guide functional analysis of the T6SS-5 is absent. In addition, the host cellular location where T6SS-5 effector(s) are targeted to remains to be determined. These facts led us to investigate the role of host factors to gain insight into the mechanism underlying cell-cell fusions stimulated by the T6SS-5. To this end, we focused on cell surface proteins and cholesterol which are of critical importance in viral induced membrane fusions during virus entry or cell-cell fusion [30–34]. We found that acute depletion of host cell surface proteins and cholesterol from macrophages infected with *B*. *thailandensis* decreased T6SS-5 induced host cell fusions.

The involvement of host cell surface proteins in MNGC formation was probed by proteolytic treatment of macrophages infected with *B*. *thailandensis* using trypsin. Similar assays were performed to analyze the nature of the receptor of dengue and Ebola virus using trypsin and proteinase K incubation of target cells, respectively [35, 36]. Proteolytic depletion of surface proteins of infected macrophages markedly reduced MNGC formation ability of *B*. *thailandensis* indicating that T6SS-5 mediated membrane fusion requires a trypsin-sensitive protein(s) on the host cell. Likewise, the depletion of surface proteins specifically on infected donor cells or uninfected recipient cells decreased the number of cell-cell fusions. Whether the T6SS-5 is used by the bacteria to puncture the plasma membrane for delivery of effector proteins to the extracellular milieu or into the neighboring uninfected cell is currently unknown. Another possible scenario is that *Burkholderia* injects T6SS-5 effector proteins directly into the cytoplasm of the host cell they infect. The potential involvement of a host protein(s) on the cell surface in MNGC formation suggests that it functions as a target or receptor of the T6SS-5 which might indicate exposure of T6SS-5 effector(s) to the external milieu. In this case, the effect observed for depletion of proteins on infected donor cells could be ascribed to degradation of the T6SS-5 effector protein(s) during trypsin treatment. Another possibility is that the T6SS-5 acts from the inside of the host cell on a surface exposed target. However, depletion of surface proteins on uninfected recipient cells caused a significant reduction in MNGC formation compared with the internal control EDTA. This result indicates that host cell protein(s) contribute to the fusion process. A potential role of one or more host surface proteins as a target of the T6SS-5 has to be elucidated in future studies. In fact, specific interactions of viral glycoproteins with cell surface receptors such as the binding of the HIV Env gp120 subunit with its primary host receptor CD4 is necessary to stimulate membrane fusion [37, 38].

The treatment of RAW264.7 macrophages with 10 mM MβCD for 1 h reduced cellular cholesterol levels by approximately 50%. This result is in agreement with a previous study reporting a 40% decrease in cholesterol levels of the same cells upon incubation with 10 mM MβCD for 30 min [39]. MβCD treatment of macrophages during infection with *B*. *thailandensis* decreased the number of cell-cell fusions by 32% suggesting that membrane fusion mediated by the T6SS-5 is susceptible to cholesterol depletion. However, we were unable to test the effect of cholesterol depletion of uninfected recipient cells as the experimental procedure resulted in a loss of viable recipient cells. Thus, whether physiological levels of cholesterol of both the recipient and donor cell are important for membrane fusion induced by the T6SS-5 remains to be determined.

To corroborate the role of cholesterol in MNGC formation we increased cholesterol levels by exogenous addition of cholesterol to the host cells at the time of infection. While this treatment did not exert adverse effects on the viability of macrophages and bacteria it diminished host cell fusion. In contrast, the exogenous addition of cholesterol did not influence MNGC formation of macrophages induced by IL-4. Together with the data on MβCD treatment these results suggest that T6SS-5 stimulated MNGC formation is influenced by cholesterol and, furthermore, that a certain cholesterol level is necessary for optimal MNGC formation. The latter is in line with a previous study demonstrating that both MβCD-based depletion of cholesterol and enrichment of cholesterol of host cells decreases internalization of *Mycobacterium smegmatis* into host cells [40]. Although entry of *M*. *smegmatis* into host cells does not involve membrane fusion it is based on the interaction of the bacteria with host surface proteins [41]. The authors of this study propose that membrane receptors implicated in *M*. *smegmtatis* entry require an optimal cholesterol content for conformation and function. Likewise, the effect of varying cholesterol concentrations on *B*. *thailandensis-*stimulated MNGC formation might be due to the interaction of a T6SS-5 protein with a host receptor that is located in cholesterol enriched membrane rafts or directly interacts with cholesterol. This assumption would explain the requirement of proteins on the host cell surface and optimal cholesterol levels for T6SS-5-induced MNGC formation. Indeed, it has been shown that the HIV gp120 receptor CD4 is associated with cholesterol enriched domains [42]. Additionally, it is conceivable that T6SS-5 effector protein(s) are targeted to cholesterol enriched domains and that altering cholesterol levels perturbs insertion or conformation of the protein(s). Assuming that T6SS-5 mediated cell-cell fusion involves bending of both membranes cholesterol could also affect MNGC formation by modulating firmness, fluidity or intrinsic curvature of the plasma membrane [43, 44].

## Methods

### Bacteria, cell line and growth conditions

*B*. *thailandensis* wild type strain E264 and *Δ*tssK*-5* (ΔBTH_II0857) mutant were grown in LB medium with shaking at 37°C [45]. The *Δ*tssK*-5* mutant harbors a non-functional T6SS-5 and was constructed and complemented in a previous study [16]. The mutant is unable to induce MNGC formation [23] (S1 Fig). For the sake of clarity, we will refer to this mutant as ΔT6SS-5 throughout the manuscript. The adherent murine RAW 264.7 macrophage cell line (DSMZ) was grown in high glucose (25 mM) DMEM medium (Gibco) supplemented with 10% FBS (Sigma) and 1 mM sodium pyruvate (Gibco) and the human monocyte cell line THP-1 was cultivated in RPMI 1640 (Gibco) supplemented with high glucose (25 mM), 10 mM HEPES, 1 mM sodium pyruvate and 10% FBS (Sigma) at 37°C with 5% CO_2_.

### Quantification of MNGC formation

MNGCs were defined as cells containing a minimum of three nuclei and quantified per field using the formula: (number of nuclei within MNGCs/total number of nuclei) × 100. MNGC formation efficiency was compared to an internal negative control treatment for each experiment.

### Surface protein depletion of infected RAW 264.7 macrophages

5×10^5^ RAW 264.7 macrophages were seeded into wells of a 6 well plate 24 h prior to the infection. Overnight cultures of *B*. *thailandensis* E264 wild type or ΔT6SS-5 mutant were washed once with PBS followed by opsonization in 10% normal mouse serum for 30 min. The bacteria were added at an MOI of 50 to the macrophages and the infection was allowed to proceed for 3 h. Next, cells were washed once in PBS and 0.05% (w/v) TPCK treated bovine trypsin (Sigma) was added to the wells. As a control, cells were treated with 0.05% (w/v) EDTA (Biochrom), which leads to cellular detachment like trypsin but lacks proteolytic activity. In addition, this control is necessary to compare the effect of trypsin as we would not observe 100% MNGC formation in the absence of any treatment. After incubation for 30 min at 37°C, complete DMEM medium was added to all cells, the detached cells were counted, centrifuged and resuspended in 0.1% (w/v) agarose (gelling temperature (1.5%): 34.5–37.5°C) (Lonza) in complete DMEM medium. Trypsin and EDTA treated cells were seeded in a 24 well plate at a density of 6×10^5^ and 4×10^5^ cells/well, respectively and spun at 400 g. At 2, 5 and 8 h post seeding, five randomly sampled images of live cells per well were taken using a Nikon Eclipse TS100 microscope and a 20× objective. Two independent experiments were performed in duplicate (N = >5000 nuclei). The viability of RAW264.7 macrophages was determined using trypan blue exclusion (N = >1000 cells).

### Surface protein depletion of specifically infected (donor) and uninfected (recipient) RAW264.7 macrophages

To analyze the role of surface proteins on uninfected (recipient) host cells, 1.5×10^5^ RAW264.7 macrophages were seeded into a 24 well plate and incubated overnight. Two petri dishes were also seeded with 2×10^6^ cells. The next day the cells in the 24 well plate were infected with opsonized *B*. *thailandensis* at an MOI of 33 and incubated for approximately 6 h. At this point the cells were washed twice with PBS and fresh media was added containing 100 μg/ml imipenem. The untreated and uninfected macrophages in the petri dish were washed once with PBS and 0.05% trypsin or 0.05% EDTA was added. After 30 minutes of incubation at 37°C the detached cells were removed and counted. These cells were then spun down at 400 *g* for 5 min after which they were diluted to 1×10^6^ cells/ml in complete medium containing 0.1% agarose. The infected macrophages were washed twice with PBS. Next, 300 µl of the trypsin or EDTA treated cells were added to the infected cells in the 24 well plate to yield approximately 6×10^5^ cells per well. The mixture of infected donor cells with uninfected recipient cells at equal rations reduced the number of infected cells. We used an MOI of 33 for all donor cell infections to yield conditions comparable to infection and treatment of all cells performed at MOI 17 as described below. The cells were spun at 400 *g* for 5 min. For the depletion of proteins specifically on the surface of infected (donor) host cells, 5×10^5^ RAW264.7 macrophages were seeded into a 6 well plate and 2×10^6^ cells were seeded in a petri dish and incubated overnight. The next day the cells in the 6 well plate were infected with opsonized *B*. *thailandensis* at an MOI of 33. At 6 h post-infection the cells were washed twice with PBS and fresh media was added containing 100 μg/ml imipenem. After 1 h the media was removed, the cells washed twice with PBS and incubated with 0.05% trypsin or 0.05% EDTA for 30 min at 37°C. During this incubation the untreated and uninfected macrophages in the petri dish were counted. Complete medium was added to the EDTA and trypsin treated cells and the detached cells were removed and counted. The EDTA and trypsin treated as well as the untreated cells were adjusted to 2×10^6^ cells/ml each. The untreated cells were mixed with EDTA or trypsin treated infected cells each at equal ratios. The cell suspensions were spun down and resuspended in 1 ml 0.1% agarose in complete media, respectively. 6×10^5^ cells (300 μl) were added to wells of a 24 well plate and spun down. After 3–4 h of incubation MNGC formation was quantified as described above based on three random fields per well. Two to three independent experiments were performed in duplicate (N = >5000 nuclei).

### Flow cytometry of trypsinized RAW 264.7 macrophages

RAW264.7 macrophages were grown to 70–80% confluence in petri dishes, washed and treated with 0.05% (w/v) trypsin or 0.05% (w/v) EDTA for 30 min at 37°C. Complete DMEM medium was then added and detached cells were counted. To detect integrin β-2 (CD18) by flow cytometry, 1×10^6^ cells per staining were resuspended in PBS containing 1% FBS. To prevent non-specific labeling, FcγII/III receptors were blocked for 15 min at 4°C using supernatant of the hybridoma 2.4G2 clone. Subsequently, cells were washed twice in PBS with 1% FBS and stained using APC-conjugated Rat Anti-Mouse CD18 antibody (BD Biosciences) for 30 min on ice in the dark. Finally, cells were washed twice in PBS containing 1% FBS and examined using a LSR II flow cytometer (Becton Dickinson) counting 2–3×10^5^ events. Data were analyzed using FlowJo 10.2 (Tree Star). Two independent experiments were performed in duplicate.

### Cholesterol depletion of infected RAW264.7 macrophages

3×10^5^ RAW264.7 cells were seeded into wells of a 24 well plate and infected the next day with opsonised *B*. *thailandensis* wild type or ΔT6SS-5 mutant at an MOI of 17. When MNGCs started to form at approximately 8 h, 10 mM methyl-β-cyclodextrin (MβCD) (Sigma; suspended in water) was added to the cells or the cells were left untreated. MβCD treatment does not result in cellular detachment. After incubation for 1 h, three randomly sampled images of live cells were taken of each well and MNGC formation was quantified as described above. Two to four independent experiments in duplicate were performed (N = >5000 nuclei). The viability of MβCD treated RAW264.7 macrophages was determined using trypan blue exclusion (N = >1000 cells).

### Cholesterol depletion of infected donor RAW264.7 macrophages

1.5×10^5^ RAW264.7 macrophages per well were seeded into a 24 well plate and infected with opsonised *B*. *thailandensis* at an MOI of 33 the next day. The infection was allowed to proceed until small MNGCs began to form. The cells were then washed twice with PBS and complete DMEM supplemented with 100 μg/ml imipenem and 10 mM of MβCD was added for 1 h. As a control, infected cells were subjected to the same procedure but incubated in the absence of MβCD. The cells were again washed twice with DPBS and media containing 100 μg/ml imipenem was added. To these cells 3×10^5^ untreated and uninfected RAW264.7 macrophages per well were added and centrifuged at 400 *g* for 5 min to bring them into contact with the other cells. After 2–3 h, three random images per well were taken and MNGC formation was determined as described above. Two independent experiments were performed in duplicate (N = >5000).

### Addition of cholesterol to infected RAW264.7 macrophages

3×10^5^ RAW 264.7 macrophages were seeded into wells of a 24 well plate and incubated overnight. The cells were infected with opsonised *B*. *thailandensis* wild type or ΔT6SS-5 mutant at an MOI of 17 and 4 μg/ml SyntheChol (Sigma; diluted in water) was added at the same time. The cells were incubated for 10 h after which images of live cells were taken to quantify MNGC formation as described above. Two to three independent experiments were performed in triplicate (N = >5000 nuclei). To determine the number of intracellular *B*. *thailandensis*, macrophages were washed with PBS and treated with 100 μg/ml imipenem in complete DMEM from 8 to 10 h post-infection. Next, the cells were washed with PBS and lysed with 1% Triton X-100 for 5 minutes. The CFU/ml was quantified by plating serial dilutions of the lysates onto LB agar based on two independent experiments performed in duplicate. Live/dead staining of RAW264.7 macrophages was performed using trypan blue exclusion (N = >1000 cells).

### Quantification of cellular cholesterol

1×10^6^ RAW 264.7 macrophages were seeded into wells of a 6 well plate. Following overnight incubation the cells were treated with 10 mM MβCD for 1 h or with 4 μg/ml cholesterol (SyntheChol; Sigma) for 10 h. Untreated cells were used as control. The cell monolayers were washed once with PBS, dried and incubated in 1 ml isopropanol per well overnight at room temperature. The supernatant containing the extracted lipids was dried under vacuum and resuspended in reaction buffer provided with the Amplex Red Kit (Invitrogen). Cholesterol levels were determined fluorimetrically utilizing the Amplex Red Kit (Invitrogen) according to the manufacturer`s instructions and normalized to macrophage protein content. The remaining cell monolayers were solubilized in 1.5 ml/well 0.1 M NaOH and the total protein content was measured using the BCA assay. Two independent experiments were performed in duplicate.

### Cytokine induced MNGC formation

THP-1 cells were seeded at a density of 2×10^5^ cells/well in 24 well plates and incubated in the presence of 120 ng/ml PMA (phorbol 12-myristate 13-acetate). After 24 h, fresh RPMI 1640 was added supplemented with 50 ng/ml human IL-4 (Sigma) or 50 ng/ml IL-4 and 4 μg/ml SyntheChol (Sigma). The medium was replaced every day with fresh medium containing the appropriate supplements. After three days the cells were fixed with methanol and stained with Giemsa (Sigma) and five randomly selected fields per well were acquired to determine MNGC formation. Two independent experiments were performed in triplicate (N = >7500 nuclei).

### Statistical analysis

Data are expressed as mean values + standard deviation (SD) of all independent experiments (except for flow cytometry data). The selection of statistical tests to compare differences between two means of unpaired samples was as follows: unpaired Student’s t test for normal distribution of data and equal variance between samples, Welch’s t test for normal distribution of data and unequal variance between samples and Mann Whitney test for non-normal distribution of data. The differences calculated by the t test and Welch’s t test were confirmed by the Mann Whitney t test except for the data shown in Fig 2D. The effect size was determined using Hedges’ *g* (normal data distribution, equal variance), Glass’s Δ (normal data distribution and unequal variance) and Mann Whitney *r* (non-normal distribution of data) following the interpretation: 0.2, small effect; 0.5, medium effect and >0.8, large effect. A *P* value of *P* <0.05 was considered statistically significant.

## Supporting information

S1 Fig The *B*. *thailandensis* ΔT6SS-5 mutant is unable to induce MNGC formation in untreated RAW264.7 macrophages. Representative images of macrophages infected with *B*. *thailandensis* wild type (wt) and ΔT6SS-5 mutant at MOI 10 for 17 h and stained with Giemsa.
(TIF)

S2 Fig MNGC formation induced by the T6SS-5 is reduced by acute proteolytic treatment. A. Representative live cell images of RAW264.7 macrophages infected with *B*. *thailandensis* wild type at MOI 50 for 3 h followed by trypsin or EDTA treatment for 30 min and agarose overlay assays of detached cells. B. Representative live cell images of live macrophages infected with *B*. *thailandensis* wild type or ΔT6SS-5 mutant as described in A. and treated with trypsin for 30 min.
(TIF)

S3 Fig Acute proteolytic treatment of infected (donor) or uninfected (recipient) host cells decreases MNGC formation. A. Representative live cell images of macrophages that were infected with *B*. *thailandensis* wild type at MOI 33 for approximately 6 h and treated with trypsin or EDTA for 30 min and mixed at equal ratios with uninfected and untreated cells at 3–4 h post seeding. B. Representative live cell images of macrophages infected with *B*. *thailandensis* at MOI 33 for approximately 6 h and mixed with uninfected cells that were treated with trypsin or EDTA for 30 min at equal ratios. Images were acquired at 3–4 h post seeding using agarose overlay assays.
(TIF)

S4 Fig MNGC formation stimulated by the T6SS-5 is affected by MβCD and cholesterol treatment during infection. Shown are representative live cell images. A. MNGC formation of macrophages infected with *B*. *thailandensis* at MOI 17 for approximately 8 h and subsequent treatment with 10 mM MβCD for 1 h or left untreated. B. Images of macrophages infected with *B*. *thailandensis* wild type or ΔT6SS-5 mutant and treated with MβCD as dscribed in A. C. Images of macrophages infected with *B*. *thailandensis* wild type at MOI 33 for approximately 8 h and mixed at equal ratios with uninfected and untreated macrophages. Images were taken 2–3 h post seeding. D. Images of macrophages infected with *B*. *thailandensis* at MOI 17 for 10 h and treated with 4 μg/ml cholesterol at the same time. E. Images of macrophages infected with *B*. *thailandensis* wild type or ΔT6SS-5 mutant and treated with cholesterol as described in D.
(TIF)

S5 Fig Representative images of MNGC formation of THP-1 cells stained with Giemsa. The cells were differentiated with PMA and incubated in the presence of 50 ng/ml IL-4 alone or 50 ng/ml IL-4 and 4 μg/ml cholesterol for 3 days.
(TIF)
