# Supplementary figures and images for: Cholesterol and host cell surface proteins contribute to cell-cell fusion induced by the Burkholderia type VI secretion system 5
Source: PLoS One. 2017 Oct 3;12(10):e0185715. doi: 10.1371/journal.pone.0185715 (PMC5626464; doi:10.1371/journal.pone.0185715)

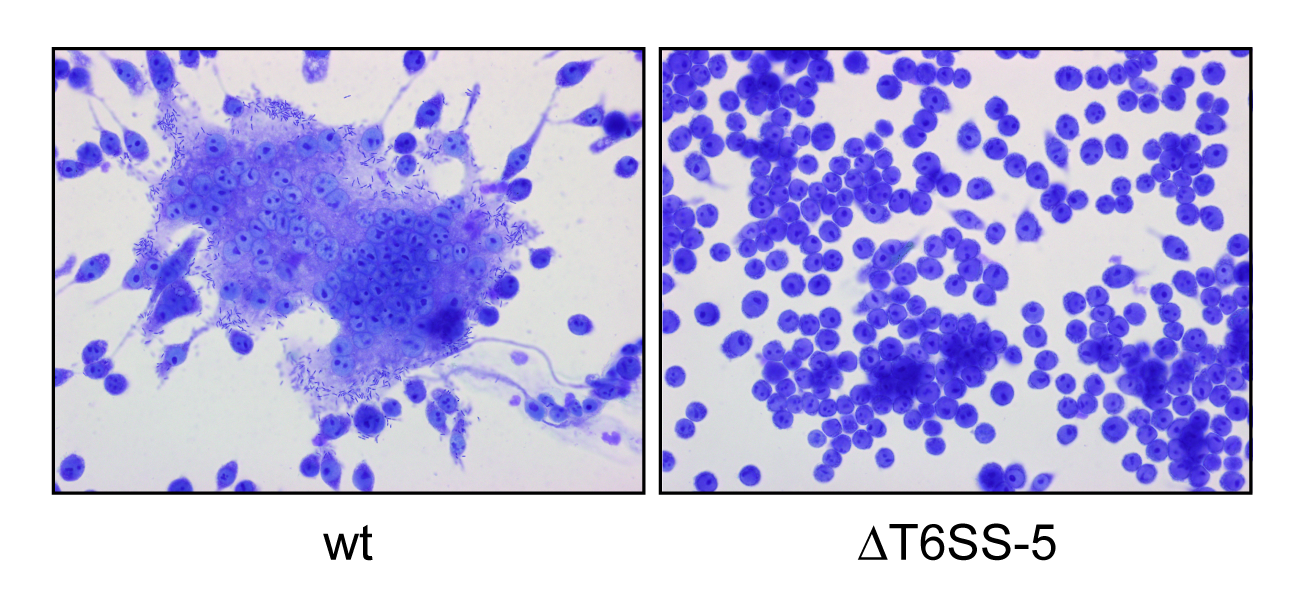

Supplement: S1 Fig — Representative images of macrophages infected with B. thailandensis wild type (wt) and ΔT6SS-5 mutant at MOI 10 for 17 h and stained with Giemsa. (TIF) [file pone.0185715.s001.tif]

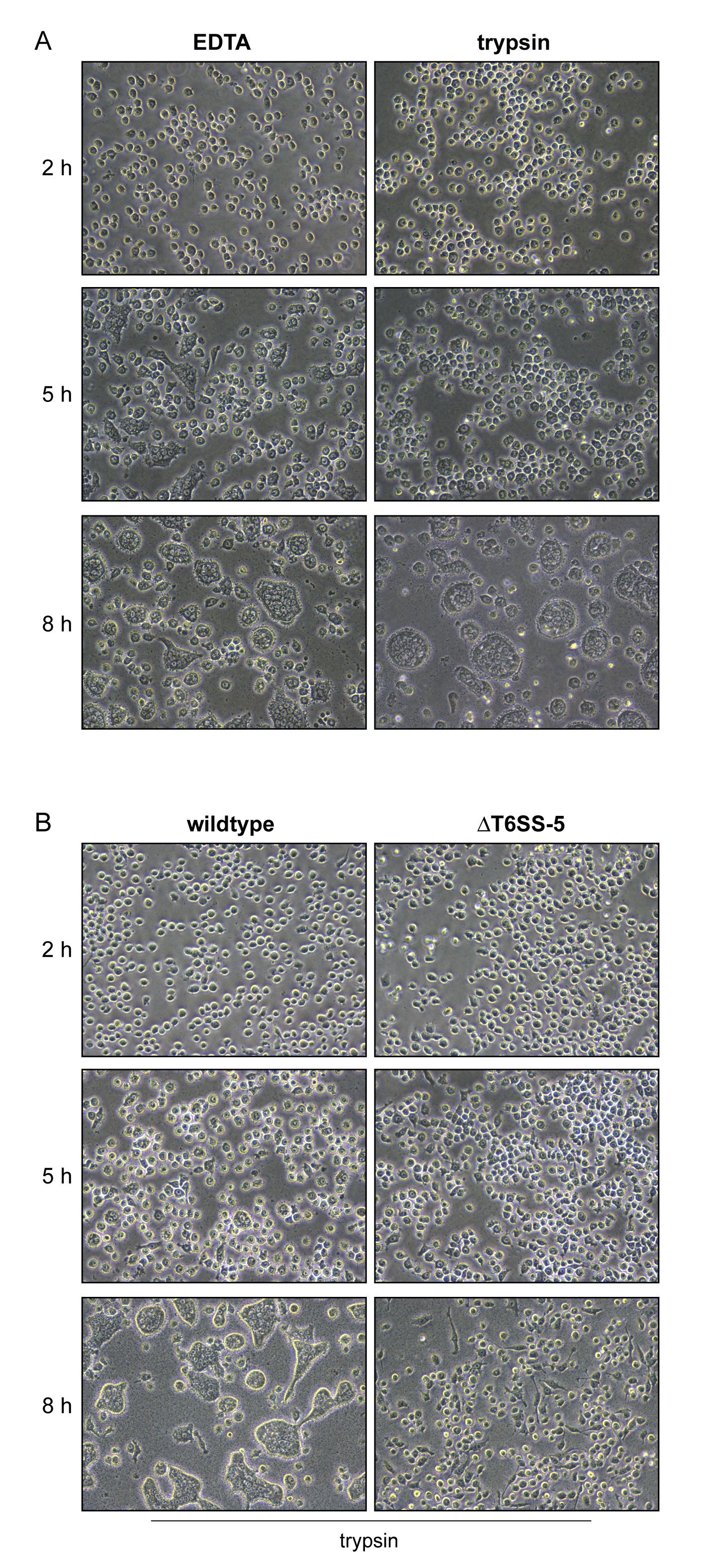

Supplement: S2 Fig — A. Representative live cell images of RAW264.7 macrophages infected with B. thailandensis wild type at MOI 50 for 3 h followed by trypsin or EDTA treatment for 30 min and agarose overlay assays of detached cells. B. Representative live cell images of live macrophages infected with B. thailandensis wild type or ΔT6SS-5 mutant as described in A. and treated with trypsin for 30 min. (TIF) [file pone.0185715.s002.tif]

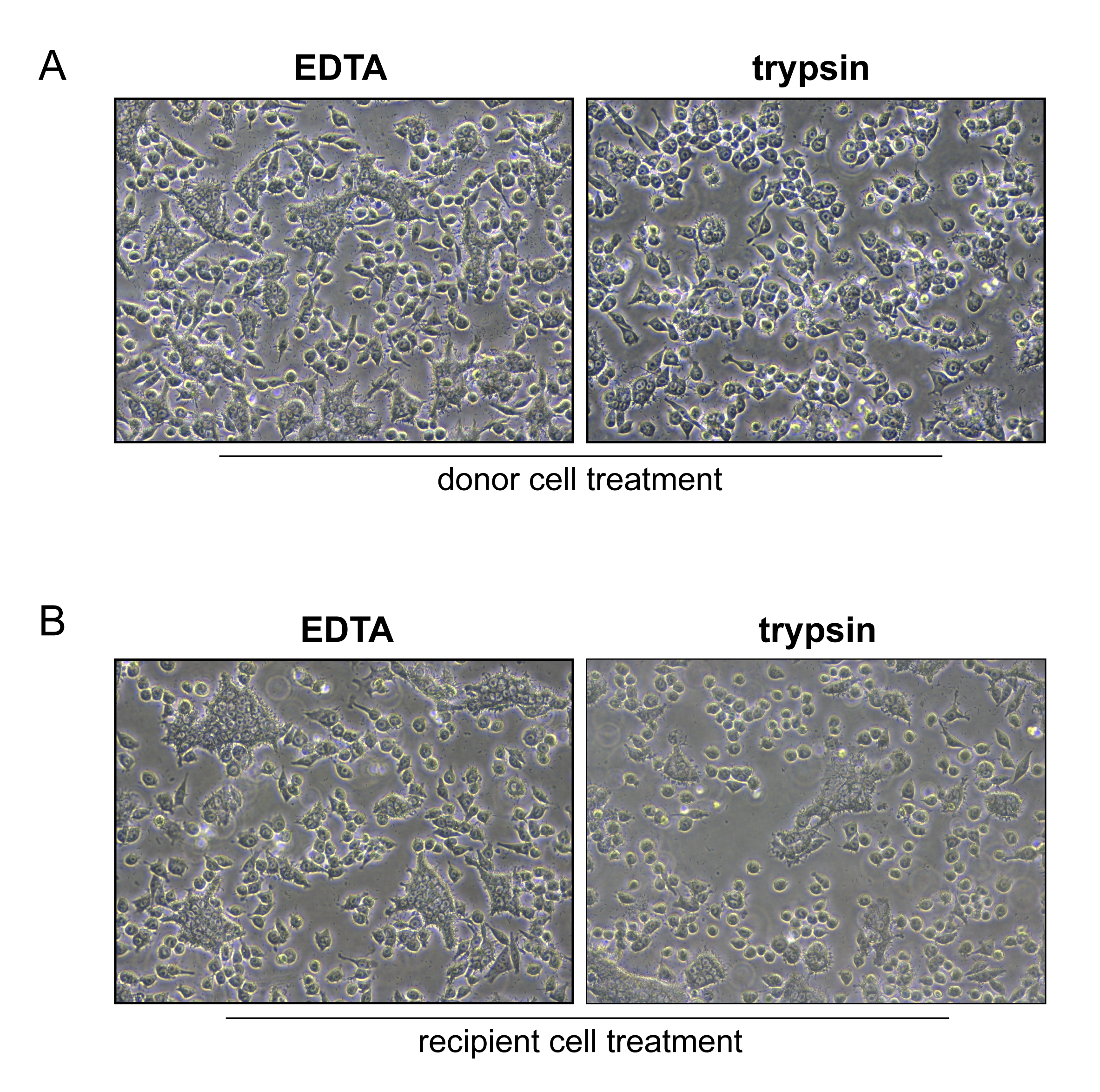

Supplement: S3 Fig — A. Representative live cell images of macrophages that were infected with B. thailandensis wild type at MOI 33 for approximately 6 h and treated with trypsin or EDTA for 30 min and mixed at equal ratios with uninfected and untreated cells at 3–4 h post seeding. B. Representative live cell images of macrophages infected with B. thailandensis at MOI 33 for approximately 6 h and mixed with uninfected cells that were treated with trypsin or EDTA for 30 min at equal ratios. Images were acquired at 3–4 h post seeding using agarose overlay assays. (TIF) [file pone.0185715.s003.tif]

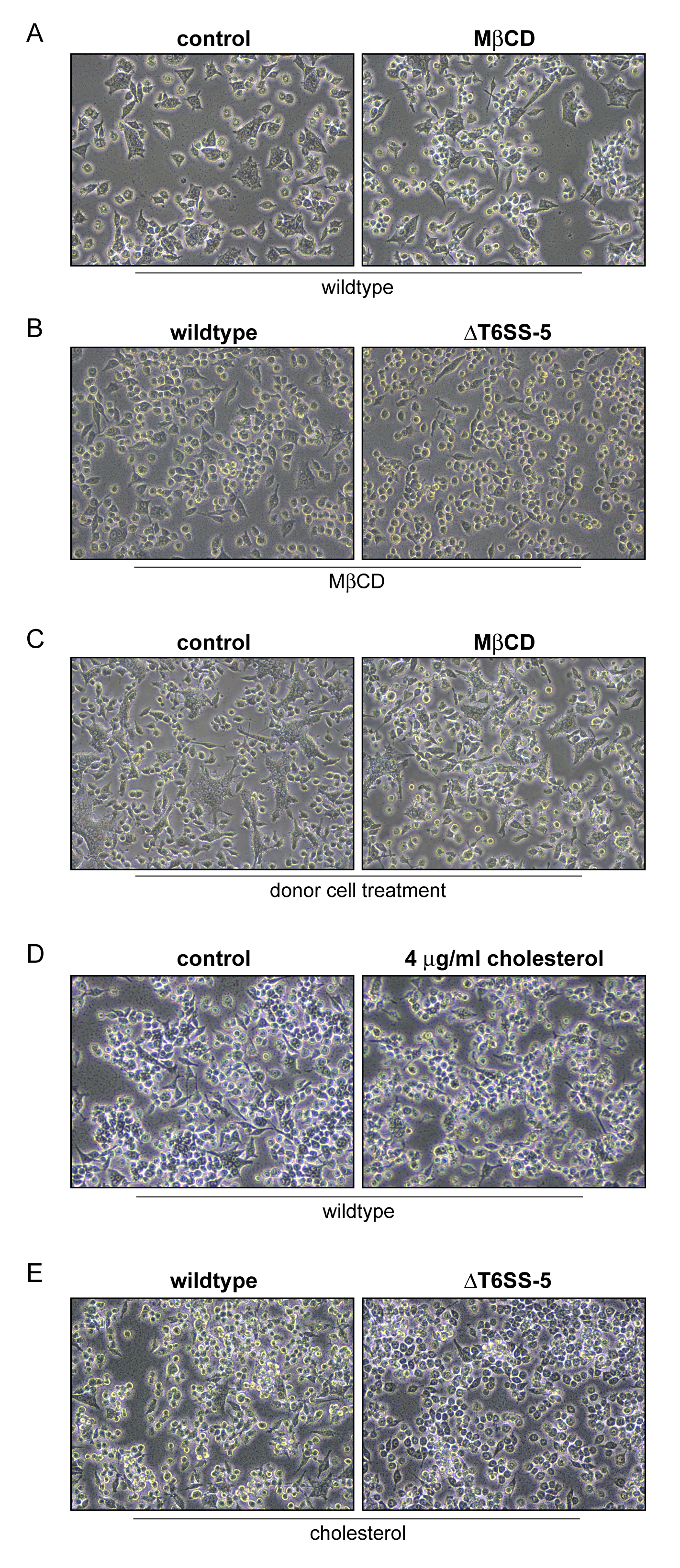

Supplement: S4 Fig — Shown are representative live cell images. A. MNGC formation of macrophages infected with B. thailandensis at MOI 17 for approximately 8 h and subsequent treatment with 10 mM MβCD for 1 h or left untreated. B. Images of macrophages infected with B. thailandensis wild type or ΔT6SS-5 mutant and treated with MβCD as dscribed in A. C. Images of macrophages infected with B. thailandensis wild type at MOI 33 for approximately 8 h and mixed at equal ratios with uninfected and untreated macrophages. Images were taken 2–3 h post seeding. D. Images of macrophages infected with B. thailandensis at MOI 17 for 10 h and treated with 4 μg/ml cholesterol at the same time. E. Images of macrophages infected with B. thailandensis wild type or ΔT6SS-5 mutant and treated with cholesterol as described in D. (TIF) [file pone.0185715.s004.tif]

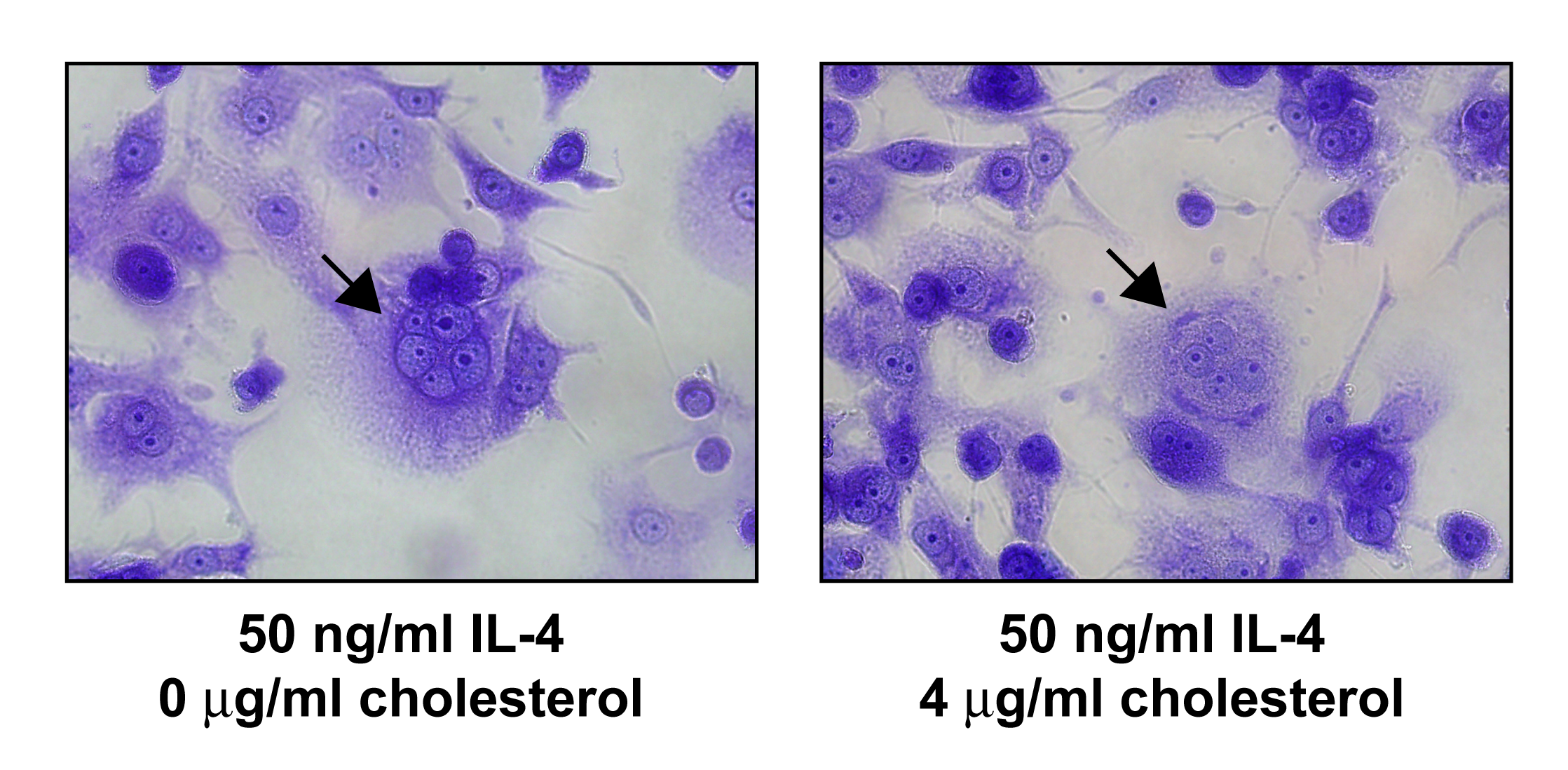

Supplement: S5 Fig — The cells were differentiated with PMA and incubated in the presence of 50 ng/ml IL-4 alone or 50 ng/ml IL-4 and 4 μg/ml cholesterol for 3 days. (TIF) [file pone.0185715.s005.tif]
